# Probiotics in Human Health: Current Evidence, Mechanism of Action, and Future Perspectives

**DOI:** 10.1155/ijm/1721210

**Published:** 2026-08-24

**Authors:** Guesh Mulaw, Gebrekidan Kidanemariam, Mouslim Bara, Teklemichael Tesfay

**Affiliations:** ^1^ College of Natural and Computational Sciences, Department of Biology, Aksum University, Aksum, Tigray, Ethiopia, aku.edu.et; ^2^ Applied Zoology Research Lab. (LZA), University Abderrahmane Mira, Bejaia, Algeria

**Keywords:** functionality, human health, physiology, probiotics, therapeutic applications

## Abstract

Probiotics are widely recognized for their potential to promote human health through diverse mechanisms that influence host physiology and microbial ecology. This review synthesizes current evidence on the biological functions, mechanisms of action, and therapeutic applications of probiotics. A comprehensive narrative literature review was conducted, with 143 records identified, screened, and assessed for eligibility before inclusion in the final analysis. The available evidence indicates that probiotics exert their beneficial effects through modulation of gut microbiota composition, enhancement of intestinal barrier integrity, competitive exclusion of pathogens, production of antimicrobial metabolites, and regulation of innate and adaptive immune responses. Clinical and experimental studies further suggest potential benefits in the prevention or management of antibiotic‐associated diarrhea, lactose intolerance, allergic diseases, hypercholesterolemia, colorectal cancer, neurological disorders, and heavy metal toxicity. Despite promising findings, probiotic efficacy remains strain‐specific and influenced by host characteristics, dosage, and treatment duration. Challenges related to strain selection, safety assessment, and standardization continue to limit broader clinical application. Overall, this narrative review provides an integrated overview of current knowledge on probiotic functionality, highlights emerging therapeutic opportunities, and identifies key research gaps that should be addressed to support evidence‐based probiotic use.

## 1. Introduction

The history of probiotics began with the history of man [1]. The health‐promoting properties of yogurt were first widely recognized through the work of the Russian scientist Elie Metchnikoff at the Pasteur Institute in Paris. He proposed that lactic acid bacteria (LAB) present in yogurt help counteract harmful putrefactive bacteria in the intestine, thereby contributing to improved gut health [2]. At the same time, Henry Tissier isolated *Bifidobacterium* species from the feces of breastfed infants and observed that these bacteria constituted a dominant component of the normal human intestinal microbiota [3]. Tissier advised giving *Bifidobacteria* to infants who had diarrhea because he thought the bacteria would replace the putrefactive bacteria that cause stomach troubles and reassume their position as the pre‐eminent intestinal microorganisms [4].

Probiotics are live bacteria that promote health by supporting the digestive and immune system, and they are used to refer to microbes that have positive effects on both humans and animals [5]. Probiotics are reported as the representative group of microorganisms associated with traditional fermented foods [6]. Prebiotics are nondigestible dietary carbohydrates that selectively stimulate the growth and activity of beneficial gut microorganisms. Synbiotics combine probiotics and prebiotics to synergistically enhance intestinal health and immune function. In addition, postbiotics, which consist of bioactive compounds produced during probiotic fermentation, have been recognized for their diverse health‐promoting properties [7]. Besides, consuming both prebiotics and probiotics (synbiotics) simultaneously may increase the survival of advantageous bacteria during their transit through the upper gastrointestinal system, as a result, they have a bigger positive effect on germs that have already colonized the intestines [8, 9].

Early‐life probiotic interventions that enhance gut resilience in infants represent a promising approach to reducing the emergence and dissemination of antibiotic resistance. Probiotic‐driven *Bifidobacterium* colonization can modulate the infant gut microbiota, resulting in a reduced abundance of antibiotic resistance genes and a lower antibiotic resistance burden [10]. The effects of prebiotic, probiotic, or synbiotic supplementations on parameters feed and water intake, weight gain, feed conversion ratio, performance index, digestibility, small intestine morphometry, and carcass traits were significantly affected on synbiotic supplementation compared with prebiotic, probiotic, and control [11]. The definition of probiotics has changed in tandem with the development of our understanding of the mechanisms underlying the usage of supplements containing live bacteria. Then, in 1974, Parker made the following claim: “Organisms and substances which help to gut microbial equilibrium are probiotics” [12]. Today, the universal definition of probiotic was established by the World Health Organization (WHO) and the Food and Agriculture Organization of the United States (FAO). These two organizations defined probiotics as “live microorganisms,” which when administered in adequate amounts, have a beneficial effect on the health of the host organism [13].

The most common types of microorganisms used as probiotics are LAB and *Bifidobacteria*, although other bacteria and certain yeasts are also used [14]. LAB are associated with habitats that are rich in nutrients, such as various food products and plant materials. They can be found in soil, water, manure, sewage, and silage and can ferment or spoil food. Particular LAB inhabit the human oral cavity, intestinal tract, and vagina, where they exert beneficial effects on the human microbiota and contribute to maintaining the health of these ecosystems. They may therefore also be candidates for application as probiotics [15]. The main probiotic microorganisms used belong to the *Bifidobacterium* and *Lactobacillus* genera [16]. They occupy different ecological positions in the human gastrointestinal tract (GIT). Lactobacilli are normal inhabitants of the small intestine, whereas *Bifidobacteria* reside in the colon [17, 18]. These bacteria are “generally regarded as safe” (GRAS) because they can reside in the human body causing no harm. Some of the common probiotic microorganisms are shown in Table 1.

**Table 1:** Microorganisms used as probiotics culture.

Number	*Lactobacillus* spp.	*Bifidobacterium* spp.	Other probiotic microorganisms
1.	*L. acidophilus*	*B. adolescentis*	*Bacillus subtilis*
2.	*Lacticaseibacillus casei*	*B. bifidum*	*Aspergillus oryzae*
3.	*Lacticaseibacillus paracasei*	*B. breve*	*Saccharomyces cerevisiae* var. *boulardii*
4.	*L. delbrueckii*	*B. longum*	*S. cerevisiae*
5.	*L. helveticus*	*B. infantis*	*Escherichia coli* Nissle
6.	*Lactiplantibacillus plantarum*	*B. lactis*	*Lactococcus lactis*
7.	*Ligilactobacillus gasseri*	*B. animalis*	*Propionibacterium freudenreichii*
8.	*Lacticaseibacillus rhamnosus*	*B. essensis*	*Leuconostoc mesenteroides*
9.	*Ligilactobacillus johnsonii*	*B. laterosporus*	*Pediococcus acidilactici*
10.	*Limosilactobacillus reuteri*		*Enterococcus faecium*
11.	*Limosilactobacillus fermentum*		*Streptococcus thermophilus*

*Note:* Source: [14].

Early‐life microbial exposures are essential for the normal growth of human physiology [19]. The ability of probiotics to modify the immunological response of the host, antagonize pathogenic microbes, or compete for adhesion sites with pathogenic microorganisms is related to the action of probiotics against microorganisms [20]. Gut microorganisms are essential for the proper development of the host immune system. Probiotics may improve intestinal immune responses by altering the composition and functional activity of the gut microbiota, thereby promoting immune homeostasis [21]. Besides, probiotics are the good microbes naturally found in the human stomach that keep our digestive health balanced. Probiotics are significant because they have both an application in industrial product development and a favorable influence on human health [22]. Besides, LAB population in gut microbiota of broilers affected nondetectable population of *C. perfringens* [23].

Given the expanding interest in microbiome‐based therapies, a comprehensive synthesis of current knowledge on probiotics is needed. Therefore, this narrative review is aimed at critically examining the current evidence on the mechanisms of action and health‐promoting effects of probiotics. Particular attention is given to their roles in modulating gut microbiota composition, strengthening intestinal barrier function, inhibiting pathogenic microorganisms, and regulating immune responses. The review further explores the potential applications of probiotics in the prevention and management of human diseases, discusses current limitations, and identifies future research directions to support their effective and evidence‐based use in healthcare.

## 2. Material and Methods

### 2.1. Search Strategy

A comprehensive narrative literature review was conducted to synthesize current evidence regarding the mechanisms of action, therapeutic applications, and future perspectives of probiotics in human health. Relevant studies were retrieved from the Scopus, Web of Science, and Elsevier databases using combinations of keywords linked by Boolean operators (AND and OR). The search strategy included terms such as “probiotics,” “probiotic strains,” “fermented foods,” “gut microbiota,” “16S rRNA gene metagenomics,” “intestinal barrier,” “immune modulation,” “host–microbe interactions,” “pathogen inhibition,” “animal models,” and “human health.”

### 2.2. Study Selection

A representative search string was applied during our analysis; we used keywords to extract data. All records identified through database searches were imported into a reference management system and screened for duplicates. Titles and abstracts were evaluated for relevance, followed by a full‐text assessment of potentially eligible studies. The Boolean operator used:

(“probiotics” OR “probiotic strains” OR “beneficial microorganisms”)

AND

(“human health” OR “gut microbiota” OR “intestinal health” OR “immune modulation” OR “host–microbe interactions” OR “pathogen inhibition”).

No restrictions were applied regarding publication year to ensure broad coverage of both foundational and contemporary studies. Peer‐reviewed research articles, clinical trials, experimental studies, reviews, and meta‐analyses were considered for inclusion. Publications were selected based on their scientific relevance, methodological quality, and contribution to understanding probiotic functionality and health outcomes.

Following screening and evaluation, a total of 143 references were retained for detailed analysis. Information concerning probiotic strains, mechanisms of action, microbiota modulation, antimicrobial activity, immunological effects, and clinical applications was extracted and synthesized narratively. The collected evidence was subsequently organized into thematic sections addressing probiotic biology, mechanisms of action, health benefits, safety considerations, and future research directions.

## 3. Results and Discussion

### 3.1. General Overview

**Figure 1:**
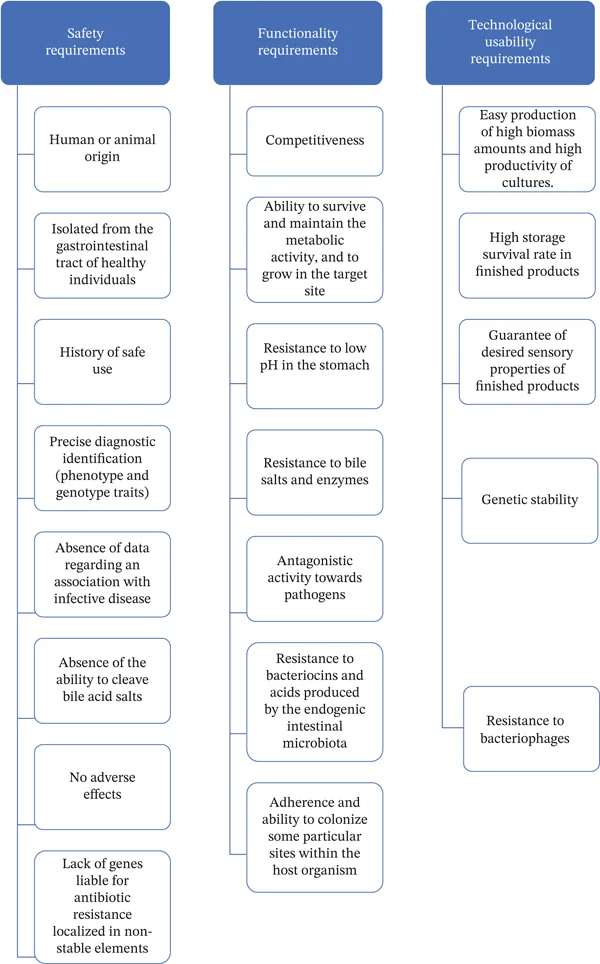
Selection criteria of potential probiotic LAB strains [26].

The selection of probiotic strains is guided by recommendations from the WHO, FAO, and the European Food Safety Authority (EFSA), which emphasize three fundamental criteria: safety, functionality, and technological suitability [24, 25] (Figure 1). Functional properties include the ability to survive passage through the GIT, adhere to intestinal surfaces, and exert immunomodulatory effects [27]. Technological requirements involve maintaining viability and functional properties during production, storage, and distribution [28]. From a safety perspective, probiotic strains should preferably originate from the healthy human gastrointestinal microbiota, possess a documented history of safe use, lack pathogenic characteristics, and transmissible antibiotic resistance genes, and should not exhibit undesirable bile salt deconjugation activity [29] (Figure 1).

To standardize probiotic evaluation, the FAO and WHO established guidelines requiring strain identification through phenotypic and genotypic characterization, comprehensive safety assessments, and validation of efficacy through well‐designed human clinical trials, preferably randomized, double‐blind, and placebo‐controlled studies [30]. In addition, probiotic candidates must withstand harsh gastrointestinal conditions, including acidic gastric environments and bile exposure, adhere to intestinal epithelial cells, and inhibit pathogen colonization through competitive exclusion, immune modulation, or antimicrobial production [31, 32].

Acid tolerance is among the primary criteria for probiotic selection because microorganisms must survive gastric transit before reaching the intestine [33]. Although gastric pH can fall below 2.0, food consumption may increase it to approximately 3.0, improving bacterial survival [34]. Numerous studies have demonstrated that LAB can tolerate acidic conditions during the relatively short gastric transit period [35–37]. Similarly, probiotic yeast isolates from traditional fermented foods showed survival rates up to 100% at pH 2.0–2.5 [38], whereas *Bacillus* spp. isolated from fermented products also exhibited high survival under acidic conditions [39].

Resistance to bile salts is another essential probiotic characteristic, as bile can disrupt bacterial membranes and reduce microbial viability [40, 41]. Physiological bile concentrations in the human intestine generally range between 0.1% and 0.5%, and concentrations of 0.15%–0.3% are commonly used for probiotic screening [32, 42] and 0.5% [43]. Several probiotic isolates, including *Lactobacillus* spp., have demonstrated strong tolerance to bile exposure, maintaining high survival rates under these conditions [44].

A key functional property of probiotics is their antimicrobial activity against pathogenic microorganisms [45]. LAB produce a variety of antimicrobial compounds, including organic acids, hydrogen peroxide, carbon dioxide, diacetyl, ethanol, and bacteriocins [46, 47]. Organic acids inhibit pathogens by lowering intracellular pH, disrupting membrane potential, and interfering with essential metabolic processes, thereby exerting broad‐spectrum antimicrobial effects against bacteria, yeasts, and molds [47, 48]. Other metabolites, such as hydrogen peroxide and diacetyl, further contribute to microbial inhibition through oxidative and metabolic mechanisms [49].

Among antimicrobial compounds, bacteriocins are particularly important due to their targeted inhibitory activity against closely related and pathogenic bacteria [50]. Nisin, produced by *Lactococcus lactis* subsp. *lactis*, is the most extensively studied bacteriocin and is approved for use in foods intended for human consumption [51]. Additional bacteriocins, including lactococcin, salivaricin, acidocin, plantaricin, and lacticin, are produced by various probiotic species and have demonstrated potential for controlling spoilage and pathogenic microorganisms in food systems [52, 53]. Consequently, bacteriocin‐producing strains have been successfully employed as starter cultures and natural preservatives to improve food safety and quality [54, 55].

The safety assessment of probiotics also includes evaluation of antibiotic resistance profiles because the global rise of antimicrobial resistance represents a significant public health concern [56, 57]. Antibiotic resistance may be intrinsic, reflecting a natural species characteristic, or acquired through mutation or horizontal gene transfer [58, 59]. Many *Lactobacillus* species exhibit intrinsic resistance to certain antibiotics, including vancomycin, due to structural differences in their cell wall peptidoglycan precursors [60–62]. Importantly, these resistance traits are generally nontransmissible and have not been associated with the transfer of resistance genes to other microorganisms, supporting the long history of safe probiotic use of strains such as *Lactobacillus rhamnosus GG* [63].

Similarly, *Bifidobacterium* species often display intrinsic resistance to several antibiotics, including nalidixic acid, aminoglycosides, and metronidazole [60, 64]. Although studies have reported variable susceptibility to vancomycin, these differences may largely reflect methodological variations in antimicrobial susceptibility testing. Therefore, careful characterization of antibiotic resistance remains an essential component of probiotic safety evaluation.

### 3.2. Mechanism of Action

**Figure 2:**
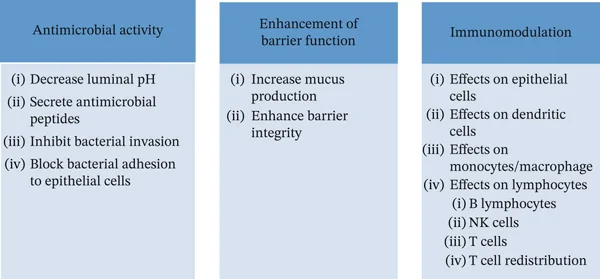
Mechanisms of action of probiotics [65].

Probiotics exert their beneficial effects through multiple mechanisms involving metabolic activity, enhancement of intestinal barrier function, modulation of the gut microbiota, and regulation of host immune responses (Figure 2). One important characteristic of LAB is their ability to produce extracellular proteinases that initiate the hydrolysis of dietary proteins, particularly milk proteins, thereby releasing amino acids essential for bacterial growth. These enzymatic activities enhance protein and fat bioavailability and increase the production of free amino acids, which may improve the nutritional status of the host, especially in individuals with reduced endogenous protease activity. Moreover, microbial proteolysis can release a variety of bioactive peptides that contribute to host health benefits [66].

Many probiotic microorganisms, including LAB and certain *Bifidobacterium* strains, produce exopolysaccharides (EPS), either as capsular polysaccharides attached to the cell surface or as extracellular polymers released into the surrounding environment [67]. Besides their industrial importance as natural biopolymers [68], EPS contribute significantly to the quality of fermented foods by acting as stabilizers, emulsifiers, viscosifiers, and gelling agents, thereby improving texture, viscosity, and reducing syneresis [69]. EPS‐producing strains belonging to the genera *Lactococcus*, *Streptococcus*, *Lactobacillus*, *Leuconostoc*, and *Pediococcus* have traditionally been used in fermented dairy products to enhance desirable rheological properties [70].

Adhesion to the intestinal mucosa is considered an important probiotic trait because it facilitates temporary colonization, prolongs persistence within the GIT, and promotes interactions with the mucosal immune system. Through adhesion, probiotics can influence gut‐associated lymphoid tissues, strengthen the intestinal barrier, and exert local and systemic immunomodulatory effects [71]. Furthermore, adhesion may contribute to competitive exclusion, a process in which probiotics inhibit pathogen attachment to intestinal epithelial cells by competing for binding sites and nutrients [72]. *In vitro* studies have demonstrated the ability of several probiotic strains, including *Lactobacillus acidophilus*, *Leuconostoc mesenteroides*, and *Enterococcus faecium*, to adhere to epithelial or abiotic surfaces and interfere with pathogen colonization [73–76]. However, despite extensive research, the exact role of adhesion in probiotic efficacy remains uncertain, as some poorly adhering strains still confer health benefits, and in vitro adhesion assays often show limited reproducibility [24].

The concept of competitive exclusion, also known as colonization resistance, is one of the most widely recognized mechanisms of probiotic action. It refers to the ability of the resident intestinal microbiota to restrict the growth and colonization of potentially pathogenic microorganisms, thereby maintaining microbial balance within the GIT. This concept was first demonstrated in the 1970s when administration of adult intestinal microorganisms protected newly hatched chicks against *Salmonella* infection [77]. Research has demonstrated that incorporating dry fermented feed fermented with *Saccharomyces* sp. into broiler diets can promote growth, improve feed conversion efficiency, increase intestinal villus height and beneficial LAB, and suppress pathogenic microorganisms in the intestine [78]. In addition to this, functional nutrition involves the regular consumption of natural or artificially derived products that regulate physiological functions and biochemical processes by maintaining or restoring the body′s microecological balance [79]. Besides, bacteria that compose the intestinal flora are influenced by the feeding habits of host animals [80].

The biological effects of probiotics are generally classified into three major modes of action [81]. First, probiotics modulate both innate and adaptive immune responses, contributing to the prevention and treatment of infectious diseases, chronic intestinal inflammation, and potentially supporting the elimination of neoplastic cells. Second, they exert direct antagonistic effects against pathogenic microorganisms while helping to maintain the balance of beneficial gut microbiota. Third, probiotics can influence microbial metabolites and host‐derived compounds, including toxins, bile salts, and dietary components, resulting in detoxification and reduced toxicity within the intestinal environment [82, 83].

At the cellular level, probiotics influence numerous components of the host immune system and intestinal barrier. They interact with epithelial cells and immune cells such as dendritic cells, macrophages, monocytes, B lymphocytes, T lymphocytes, regulatory T cells, and natural killer (NK) cells, thereby affecting both innate and adaptive immune responses. Through these interactions, probiotics contribute to maintaining intestinal homeostasis, strengthening mucosal defenses, and regulating inflammatory processes [84].

The safety of probiotic strains is increasingly assessed through whole‐genome sequencing, which complements traditional safety evaluations by identifying genes associated with virulence, antimicrobial resistance, and other potential risk factors. For example, genomic analysis of *Lactobacillus plantarum* JDM1 identified several genes associated with virulence‐related functions; however, most were classified as defensive or nonclassical factors and were not considered harmful to the host [85].

The evaluation of probiotic viability and quality traditionally relies on plate count methods, which measure the ability of bacterial cells to grow and form colonies [86]. However, these methods cannot accurately detect damaged or viable but nonculturable cells, potentially leading to underestimation of probiotic populations [87]. To overcome these limitations, culture‐independent techniques such as quantitative PCR (qPCR) and flow cytometry have been developed to assess bacterial viability more accurately and provide rapid characterization of cellular physiological states [88, 89].

More recently, postbiotics—bioactive compounds produced by probiotic microorganisms—have attracted considerable interest because they may provide health benefits similar to those of live probiotics while avoiding risks associated with viable microorganisms, such as bacteremia, transfer of virulence genes, or dissemination of antibiotic resistance determinants. In addition, postbiotics offer greater stability during storage and can remain effective at room temperature for extended periods [84].

### 3.3. Benefits of Probiotics

Probiotics contribute to human health by maintaining intestinal microbial balance and suppressing pathogenic microorganisms as well as noncommunicable disease [90, 91]. Extensive evidence indicates that probiotics enhance immune function, improve gastrointestinal health, reduce metabolic and inflammatory disorders, support neurological health, and protect against environmental toxicants [92, 93]. They are also important in alleviating the symptoms of lactose intolerance and in enhancing growth [94]. Probiotics are useful in food biodetoxification and effectively reduce food contamination [95].

#### 3.3.1. Immunomodulatory and Antimicrobial Effects

Studies conducted *in vitro*, animal models, and human clinical trials have demonstrated that probiotics can modulate host immune responses [96]. Consumption of *B. lactis* HN019 and *L. rhamnosus* HN001 has been associated with enhanced immune parameters and increased NK cell activity in elderly individuals [97–99]. These effects are mediated through stimulation of mucus production, macrophage activation, increased immunoglobulin A (IgA) secretion, neutrophil activity, and regulation of cytokine production [98].

In addition, probiotics exert antimicrobial activity through the production of short‐chain fatty acids (SCFAs), bacteriocins, organic acids, hydrogen peroxide, and other bioactive compounds that inhibit pathogen growth and survival [100]. They also enhance host antimicrobial defenses and compete with pathogens for nutrients and epithelial binding sites [100].

#### 3.3.2. Gastrointestinal and Metabolic Benefits

Probiotics have demonstrated significant benefits in gastrointestinal health, particularly in alleviating lactose intolerance and reducing antibiotic‐associated diarrhea (AAD) [101, 102]. The presence of *β*‐galactosidase–producing bacteria improves lactose digestion and reduces symptoms such as bloating, abdominal pain, and diarrhea [103]. Likewise, probiotic supplementation helps restore intestinal microbiota disrupted by antibiotic treatment, thereby reducing the incidence and severity of AAD [104].

Probiotics may also contribute to improved lipid metabolism. Several studies suggest that certain *Lactobacillus* and *Bifidobacterium* strains reduce serum cholesterol by deconjugating bile acids, promoting cholesterol excretion, and inhibiting cholesterol synthesis [104, 105]. These mechanisms may help lower cardiovascular disease risk in hypercholesterolemic individuals [105]. Our intestinal microbiota serve many roles vital to the normal daily function of the human GIT. Many probiotics are derived from our intestinal bacteria and have been shown to provide clinical benefit in a variety of gastrointestinal conditions [106, 107] (Table 2). It also possesses mechanisms to eliminate harmful agents that penetrate this barrier [109]. These physiological functions are strongly influenced by the gut microbiota, which regulate the expression of epithelial genes associated with nutrient transport and metabolism, maintenance of mucosal integrity, xenobiotic metabolism, enteric nervous system function, intestinal motility, hormonal and developmental responses, angiogenesis, and the organization of the cytoskeleton and extracellular matrix [110]. The gut microbiome plays a pivotal role in digestion, immunity, and disease resistance [111].

**Table 2:** Potential beneficial effects of probiotic supplementation against metabolic disorders.

S/no	Probiotic action	Mechanism	Physiological outcome
1.	Energy metabolism	Short‐chain fatty acids (SCFA) production, vitamin synthesis, bile acid metabolism	Improved glucose homeostasis, lipid metabolism
2.	Barrier function	Strengthened tight junctions, increased mucus secretion	Reduced intestinal permeability
3.	Immune modulation	Increased IgA, macrophage activation, anti‐inflammatory cytokines	Reduced inflammation
4.	Gut microbiota	Increased beneficial bacteria, bacteriocins, pathogen inhibition	Improved microbial diversity and colonization resistance

*Note:* Source: [108].

#### 3.3.3. Prevention of Chronic and Allergic Diseases

**Figure 3:**
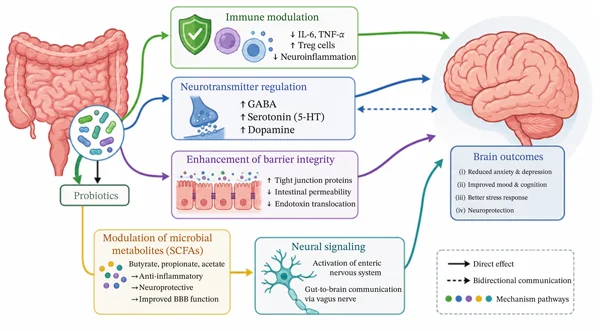
Mechanisms of probiotic action on the gut–brain axis [120].

Growing evidence suggests that probiotics may reduce the risk of colorectal cancer by modulating gut microbiota composition, suppressing carcinogenic bacterial enzymes, regulating immune responses, and reducing intestinal inflammation [112, 113]. Probiotics have demonstrated anti‐inflammatory and antitumor effects in preclinical and clinical studies [114]. Moreover, probiotic supplementation significantly decreases the occurrence of diarrhea and the frequency of daily bowel movements [115]. Dysbiosis, an imbalance in gut microorganisms, can promote cancer. Conversely, probiotics have the potential to enhance gut health and restore immunological equilibrium [116]. It is characterized by the depletion or overrepresentation of specific bacterial taxa and alterations in their metabolic activities. Therapeutic supplementation with conventional probiotics, either alone or in combination with prebiotics, as well as next‐generation probiotics (NGPs) or postbiotics, has emerged as a promising adjunctive approach to cancer treatment by restoring microbial homeostasis and modulating host physiological and immune responses [117]. The therapeutic potential of probiotics in the management of depression is supported by the mechanisms of the microbiota–gut–brain axis (MGBA), which is known to contribute to the pathophysiology of depressive disorders. Consequently, probiotics may serve as effective adjuncts to conventional therapy for major depressive disorder (MDD) and could also be considered as a stand‐alone intervention for mild MDD, offering a promising avenue for improving the treatment of depression [118]. Researchers also reported that psychobiotics are efficacious in improving neurodegenerative and neurodevelopmental disorders, including autism spectrum disorder (ASD), Parkinson′s disease (PD), and Alzheimer′s disease (AD) [119] (Figure 3). Probiotics represent a novel and promising strategy for managing neurodegeneration particularly AD and PD [121]. Certain probiotic strains have also been associated with increased microbial diversity and reduced tumor‐promoting factors in colorectal cancer patients [122, 123].

Probiotics may additionally play a role in preventing allergic disorders, particularly eczema in children [124]. Their beneficial effects are attributed to enhanced mucosal barrier function, immune regulation, reduced inflammatory responses, and improved degradation of dietary antigens. Clinical studies have reported a lower incidence of eczema among infants receiving probiotic supplementation, particularly *L. rhamnosus GG* [125]. Other studies indicate that addition of probiotics to the diet for weeks improved the immune response without the release of inflammatory cytokines, thereby reducing the onset of systemic inflammatory induced diabetes [126].

#### 3.3.4. Neuroprotective Effects and Protection Against Heavy Metal Toxicity

The gut–brain axis has emerged as an important pathway through which probiotics influence neurological health [127, 128]. Clinical studies suggest that specific probiotic strains may improve behavioral outcomes in children with ASDs and reduce symptoms of anxiety, stress, and depression [129, 130]. These effects may be mediated through modulation of gut microbiota composition and production of neuroactive compounds such as *γ*‐aminobutyric acid (GABA) [131]. The MGBA is a complex bidirectional communication system linking the GIT and the brain through neural, endocrine, and immunological signaling pathways. Perturbations within this network can have reciprocal effects on both systems, whereas dysregulation of the axis may contribute to functional abnormalities in the enteric and central nervous systems [132].

Probiotics also show promise in mitigating heavy metal toxicity. Several *Lactobacillus* and *Bifidobacterium* strains can bind cadmium and lead, reduce intestinal absorption, alleviate oxidative stress, and limit tissue accumulation of toxic metals [133, 134]. Experimental and human studies indicate that probiotic supplementation may protect against heavy metal‐induced toxicity, highlighting its potential as a dietary strategy for populations exposed to environmental contaminants [135, 136].

## 4. Future Perspectives

The increasing prevalence of microbiota that exhibit resistance and tolerance to traditional drugs and antibiotics has reduced the effectiveness of many therapeutic agents. In response, several probiotic strains have demonstrated promising health benefits, particularly through their strong anti‐inflammatory and antiallergic activities. Probiotics offer a promising preventive and therapeutic advancement [137]. Increasing evidence indicates that regular consumption of these products may contribute to the prevention and management of several health disorders, such as irritable bowel syndrome, lactose intolerance, gastroenteritis, obesity, chronic diarrhea, allergic diseases, atopic dermatitis, and infectious diseases [138]. The field of probiotics is rapidly evolving, with the development of novel microbiome‐modulating strategies, including prebiotics, synbiotics, postbiotics, microbial consortia, live biotherapeutic products, and genetically engineered microorganisms. Concurrently, increasing attention is being directed toward polyphenols, dietary fibers, and fermented foods because of their potential to promote human health [139]. However, most NGP candidates have complex nutritional requirements and are highly sensitive to oxygen, creating significant technological challenges for their large‐scale production [140]. The next generation of probiotics has become a major topic in scientific research [141].

Recent advances in high‐throughput sequencing technologies have enabled the identification of gut microorganisms with substantial health‐promoting properties, facilitating the development of NGPs allowing for more precise and customized treatment approaches [142]. By integrating synthetic biology and bioinformatics approaches, these NGPs are designed to target specific disease conditions while exhibiting improved stability and viability [143]. Improvements in metabolomic and genetic technologies have increased our understanding of how probiotics work, allowing for more precise and customized treatment approaches.

## 5. Conclusion

Probiotics have emerged as future biological elements able to promote human health through multiple mechanisms, including modulation of gut microbiota composition, enhancement of intestinal barrier function, competitive exclusion of pathogens, production of antimicrobial metabolites, and regulation of immune responses. The most extensively studied probiotic microorganisms are species belonging to the genera *Lactobacillus* and *Bifidobacterium*, although a growing number of other beneficial microorganisms are being investigated for their therapeutic potential.

This narrative review highlights the potential role of probiotics in the prevention and management of a wide range of health conditions, including gastrointestinal disorders, metabolic diseases, allergic conditions, neurological disorders, and infections. Furthermore, advances in microbiome research and molecular technologies have improved our understanding of host–microbe interactions and the mechanisms underlying probiotic functionality.

Despite these, probiotic efficacy remains highly strain‐specific and is influenced by factors such as dosage, treatment duration, formulation, and host characteristics. Future well‐designed, randomized, double‐blind, placebo‐controlled clinical trials are needed to establish standardized guidelines for probiotic use and to validate their long‐term safety and effectiveness. Overall, probiotics represent a rapidly evolving field with considerable potential to contribute to the prevention of human diseases.

## Funding

No funding was received for this manuscript.

## Conflicts of Interest

The authors declare no conflicts of interest.

## Data Availability

As this is a review paper, no additional data are available. Rather, the findings came from reviewing original research articles.
